# A New Approach for Characterizing the Intermediate Feature of α-Chymotrypsin Refolding by Hydrophobic Interaction Chromatography

**DOI:** 10.3390/ijms10020616

**Published:** 2009-02-18

**Authors:** Congyu Ke, Jianjun Li, Zhenling Liu, Xindu Geng

**Affiliations:** 1 Institute of Modern Separation Science, Shaanxi Key Laboratory of Modern Separation Science, Key Laboratory of Synthetic and Natural Functional Molecule Chemistry of Ministry of Education, Northwest University, 710069 Xi’an, P.R. China; 2 Xinxiang Medical College, Xinxiang, 453003, Henan Province, P.R. China

**Keywords:** Protein folding, protein drugs, misfolding, intermediates, protein folding liquid chromatography, characterization, hydrophobic interaction chromatography, α-Chymotrypsin, stoichiometric displacement theory

## Abstract

A new approach for characterizing the intermediate of urea-denatured α-chymotrypsin (α-Chy) by hydrophobic interaction chromatography (HIC) is presented. The contact surface region (*Z, S*), affinity (log*I*), and the character of interaction force (*j*) of the α-Chy to the stationary phase of HIC (STHIC) between the intermediate (M) and native (N) states were found to be quite different as urea concentration (*C*_urea_) changes. With the changes in *C*_urea_, a linear relationship between log*I* and Z was found to exist only for its N state, not for M state, indicating the interaction force between α-Chy in N state to the STHIC to be non-selective, but selective one for its M state. Also, the measured magnitude of both log*I* and *Z* in M state is only a fifth of that in N state. All three parameters were employed to distinguish protein in the N state from that in the M state. It would be expected that this result could be employed to distinguish any kind of non-functional protein having correct three-, or four-dimensional molecular structure from their stable M state of any kinds of proteins, and/or other proteins in proteome investigation, separation process of protein, and intensively understanding the intrinsic rule of protein folding in molecular biology.

## 1. Introduction

Scientists usually understand proteins to be more extensively in the native (N) state with normal molecular conformations than in an unfolded (U) or intermediate (M) state with abnormal protein folding molecular conformations, because protein drugs exist in N state and this is more useful for human life. However, the appearance of bovine spongiform encephalopathy (BSE) in England required scientists to investigate proteins in an abnormal (non-native) state and thus it was found that BSE disease is also dependent on the molecular conformation of proteins. The molecular conformations of protein in buffers appear flexible, but still in N state, while proteins in U and/or M states, are actually in a very stable molecular conformation.

How to prevent the formation of U and M states of a protein? Also, if both exist already, how to convert them to their corresponding N state, or how to increase the efficiency of protein folding? This has became an important field in both molecular biology and liquid chromatography (LC), and also the renaturation for producing recombinant protein drugs in biotechnology using *E coli*. According to Anfinsen’s theory [1], the three-, or four-dimensional structure of protein molecule only depends on its primary structure, or amino acid sequence. In other words, each of the N, U, and M states has the same amino acid sequence, but various molecular conformations. An intensive understanding and characterization of the nature of the U and M states would be very helpful to find a better way to accomplish this conversion from U and/or M state into the corresponding N state.

Many methods, such as optical or calorimetric methods or NMR, can be employed to investigate the character and the changes in molecular conformation over a period of time [2–4]. All of them need a pure sample. Many kinds of LC can be employed to achieve this by a chromatographic separation process. Hydrophobic interaction chromatography (HIC) was previously reported as being not only a very efficient tool for investigating protein folding, but also for protein renaturation with simultaneous purification in biotechnology and this method is called protein folding liquid chromatography(PFLC) [5].

In some cases unfolded proteins cannot, or cannot completely be refolded by HIC, so the obtained one or more M state species should form at the interface between the stationary and mobile phases and have various hydrophobic strengths, and thus they can be separated from each other during the chromatographic process. This may provide a theoretical basis and methodology for on-line isolation, capture and investigation of the M state that originally existed in a complex mixture solution, without *a priori* purification of the N and/or M state of the target proteins.

The two linear parameters log*I* and *Z*(S) (for their physical meanings, see later) of the stoichiometric displacement theory for retention (SDT-R) were widely employed for characterizing the changes in molecular conformation of proteins and molecular structure of small solutes and biopolymers [6,7]. The M and N states of a protein must have a different magnitude of log*I* and *Z* (S) [8]. This difference should be employed to distinguish them from each other.

In this study, an active protein, α-chymotrypsin (α-Chy), was selected as a standard protein (N state) and the stable intermediate of α-Chy as the typical M state of a protein. HIC was selected as a typical HPLC method for the on-line preparation and purification of the prepared stable M state of α-Chy and subsequently identifying its N and M states by optical, bioactivity, and matrix-assisted laser desorption/ionization time-of-flight mass spectrometry (MALDI-TOF MS). With the measurment and comparison of the three linear parameters of the SDT-R for α-Chy in the N and M states, a new on-line chromatographic characterization of the α-Chy in M state is established.

## 2. Principle

A protein in N state has a correct three-, or four-dimensional molecular structure that allow flexible changes in molecular conformation in buffers, and the hydrophobic amino acid residues exist inside the protein molecules. Proteins in the M and U states have a very compact molecular, or random coil molecular structure, do not present flexible changes in molecular conformation and the hydrophobic amino acid residues are exposed on the surface of protein molecules. When the three species of a same protein contact with the region of the interface of a liquid-solid, their contact surface areas, the type and magnitude of the interaction forces of the target protein with the STHIC (stationary phase of hydrophobic interaction chromatography) should be quite different.

The hydrophobicity of the surface of the LC stationary phase plays here an important role. For reverse phase liquid chromatography (RPLC), the strong hydrophobicity makes a protein partially, or completely lose its N state and convert to its U state. If the hydrophobicity is moderate, such as in HIC, the protein can not only retain its N state, but some protein which had been originally denatured can now be refolded by HIC [9]. So long as a protein in U state cannot be refolded, or only partially refolded by HIC, one, or several stable M states shall exist. When the molecular structure of the protein changes to a large extent, such as protein in the U state converts to an N state, the characterization parameters to express the changes in surface area, interaction forces, affinity of the protein to STHIC vary tremendously also. On the contrary, proteins in the U and/or M states cannot be refolded by HIC, so their molecular structure and corresponding characterization parameters will not change significantly. So long as these characterized parameters can be found and exactly measured, these different changes sourced from the molecular conformation of a protein can be employed to distinguish them from each other.

Two linear parameters of the stoichiometric displacement theory for retention (SDT-R), *Z* (*S*) and log*I* have been widely employed to express the molecular conformation and also the affinity of proteins towards stationary phases by many scientists. The topic has been reviewed [6–8]. For HIC, the expression of the SDT-R is gven by Equation (1) [10]:

(1) $$\text{log}k'=\text{log}I-Z\text{log}a_{\text{H}2\text{O}}$$

where, *k*’ is the capacity factor of protein, *Z* denotes the moles of water released from the contact surface region of one mole of solvated protein adsorbed onto the STHIC, log*I* is a constant relating to the affinity of one mole of the protein for the stationary phase. The term *a*_H2O_ represents the water activity in the mobile phase. When the HIC stationary phase and mobile phase, as well as other chromatographic conditions, such as column temperature, are given, both of *Z* and log*I* are constants and thus Equation (1) is a linear equation and easily measured by experiment by plotting log*k’ versus* log*a*_H2O_. *S*, presented as an identical parameter to *Z* by Snyder’s group, can be obtained by:

(2) $$\text{log}k'=\text{log}K_{\text{w}}+S\Phi$$

Here *K*_w_ denotes the partition coefficient of protein in stationary phase and pure water [6–8], *Φ* is the volume fraction of displacer in mobile phase. Because Equation (2) is an approximately linear equation, thus *S* is an empirical constant. The relationship between Z and S was connected by Equation (3) as [8]:

(3) Z=2.3SΦ

By using Equation (3) and the physical meaning of *Z*, both have been widely employed to characterize the changes in the molecular conformation of proteins. With Equation (3), both *S* and *Z* can be converted to each other. According to a previous report the *Z* value measured by isocratic elution is more accurate than the *S* obtained from gradient elution [11] and thus *Z* together with log*I* were employed to investigate the molecular conformation of proteins in this study.

An excellent linear relationship between log*I* and *Z* of small solutes (non-polar and polar) and proteins in N state exist [12], but it is only valid for solute retention dominated by non-specific interactions, such as RPLC and HIC. A linear relationship between log*K*_w_ and *S* was also reported, but is only valid in methanol-water as the mobile phase [6]. This relationship can be expressed as Equation (4).

(4) logI=Zj+logϕ

The physical meaning of *j* here denotes the affinity of one mole of displacer (water in HIC) to the stationary phase of LC, *ϕ* is the column phase ratio defined by thermodynamics. Because the stoichiometric displacement process between solute and solvent is a reversed process, in other words, one mole of solute displacing Z moles of solvent is equivalent to one mole of solvent displacing 1/*Z* solute. The stoichiometric parameter *j* shown in Equation (4) just describes the latter process. Thus, the physical meaning of *j* as solvent as displacer actually corresponds to that log*I* as solute as displacer. It is actually Equation (4) that has been experimentally found to have a very good linear relation for protein separation by HIC, either under various proteins at a same composition of mobile phase, or a given protein with various salts, pH, even denaturants [10]. With the new concept of molecular interaction about the origin of long-range attraction between hydrophobes in water presented by Despa *et al*. [13], this linear relationship between proteins and the stationary phase of HIC (STHIC) can be theoretically explained as a non-selective interaction that dominates protein separation in this instance. Thus, the goodness of the linearity can be employed to justify the kind of interaction forces between solute and stationary phase of LC.

## 3. Experimental

### 3.1. Equipment

The chromatographic system (Shimadzu, Japan) consists of two pumps (TC-10ATVP), a system controller (SCL-10VAP), UV-Vis detector (SPD-10AVP), constant temperature column box (CTO-10ASVP), a ClassVP5.03 chromatographic workstation and chromatographic column (silica-based, 150 mm × 4 mm I.D., stainless steel column). Other equipments are: matrix assisted laser desorption/ionization time of flight mass spectrometer (MALDI-TOF MS, Axima CFR plus, Kratos Analytical Company of Shimadzu Biotech., Manchester, Britain), UV spectrophotometer (UV-1601PC, Shimadzu) and fluorescence spectrophotometer (F-4500 fluorescence spectrophotometer, Hitachi Co., Japan) were employed to measure the molecular mass, identify the optic characteristics of the various species of α-Chy and bioactivity. HPLC grade water was prepared with a Barnstead E-Pure system (Barnstead Co. Ltd, USA). Freezing drier was a PLPHA-1-4.

### 3.2. Chemicals

The α-Chymotrypsin (α-Chy) protein standard was purchased from Sigma (St. Louis, MO, U.S.A.). The silica-based hydrophobic interaction chromatography packing (PEG-600, pore size 30 nm, particles, 6 μm) was purchased from Xi’an Aolan Scientific & Technology Co., (Xi’an, P.R. China, http://www.Aulanst.com). Ammonium sulphate and potassium dihydrogen phosphate and urea were purchased from Xi’an Chemical Reagent Corp., P.R. China (Analytical grade). Other reagents employed in this study are analytical grade, and the water is deionized water.

Mobile phase I consists of solutions A1 and B1. Solution A1: 2.5 mol/L ammonium sulphate and 0.050 mol/L potassium dihydrogen phosphate (pH 7.0). Solution B1: 0.050 mol/L potassium dihydrogen phosphate (pH 7.0). The solutions were used for the separation of the folded intermediates of α-Chy after filtration. The mobile phase II consists of solutions A2 and B2. Solution A2: 2.5 mol/L ammonium sulphate + 0.050 mol/L potassium dihydrogen phosphate + *X* mol/L urea (pH 7.0); Solution B2: 0.050 mol/L potassium dihydrogen phosphate + *X* mol/L urea (pH 7.0). The “*X*” is the concentration of urea, and these solutions were used for determination of log *I* and *Z*(S).

### 3.3. Experiment methods

#### 3.3.1. Chromatographic procedure

Solid α-Chy (6.0 mg) was dissolved into 1.0 mL of 1.0, 2.0, 3.0, 4.0, 5.0, 6.0, 7.0 and 8.0 mol/L urea solutions, respectively, and then the denatured α-Chy solutions remained standing for 24 h in room temperature. The sample solution was injected into HIC column after it was equilibrated by 100% solution A1 and then a linear gradient elution occurred from 100% solution A1 to 100% solution B1 of mobile phase I for 30 min with a delay 10 min and detection at 280 nm. Each eluted fraction in different chromatographic conditions was collected and measured for the recoveries of bioactivity and mass. The collected fraction including the intermediate of α-Chy was freeze-dried for later experimental samples.

#### 3.3.2. Measurement of UV and fluorescence emission (FE) spectrum

The sample solution including N, M in the collected fractions, and U in the unfolded solution were separated and measured by UV (195~350 nm) and FE (250 nm) with the reference solution having the same composition as the sample solution except the α-Chy.

#### 3.3.3. Measurement of log *I* and *Z* (S)

Both isocratic and gradient elutions can be employed to measure log *I* and *Z*, or *S*. The former is accurate, but needs more experiments [10], while the later is simple and fast to obtain, but the result is an approximation [8]. In this study, each of the log *I* and *Z* of α-Chy was measured by isocratic elution with flow rate 1.0 mL/min and detected at 280 nm. A suitable composition of the mobile phase II containing different urea concentrations (*C*_urea_) was adjusted by the ratio of the solution A2 to solution B2. Before each injecting, the chromatographic column was equilibrated 20 min by the mobile phase with the selected composition. The dead volume was detected by the solution of sodium nitrite. For convenience, each activity coefficient of water in both stationary and mobile phases was simply taken as unity, *i.e*., *a*_H2O_ in Equation (1) can be written as its concentration form, [*H_2_O*] (mol/L) in the mobile phase. The term [*H_2_O*] can be calculated with Equation (5) [10] as:

(5) $$[H_{2}O]=(d_{A}\Psi_{A}+d_{B}\Psi_{B}-Ws)/0.018$$

where *d_A_* and *d_B_* are the densities of solutions A and B (g/mL), respectively, *Ψ_A_* and *Ψ_B_* are the volume fractions (v/v, %,), solutions A and B, respectively, and *W_S_* is the weight of salt in a fixed volume of the mixed solution (g/mL). The dimension of 0.018 is kg/L

#### 3.3.4. Mass spectrum by MALDI-TOF-MS

The collected fractions were freeze-dried and then dissolved in ultra-water and mixed with the saturated solution of CHCA (α-cyano-4-hydroxycinnamic acid) at the ratio of 1:1 (v/v). Then the obtained 1 μL sample solution was pointed on target board and dried by air blowing. The mass analysis was carried out at the model of linear positive-ion, and the instrument was standardized by the external standard pair of the mixture of peptides and the standard sample of proteins.

#### 3.3.5. Measurement of concentration and bioactivity of α-Chy

The determination of protein concentration of α-Chy was carried out according to Ref. [14]. Bioactivity of protein was measure according Ref. [15].

## 4. Results and Discussion

Supposing the activity coefficient of the employed mobile phase under all conditions is unity and thus Equation (1) becomes its concentration form Equation(1a) as:

(1a) $$\text{log}k'=\text{log}I-Z\text{log}[H_{2}O]$$

where [*H_2_O*] is water mole concentration, mol/L.

### 4.1. Preparation, identification and stability of M state of α-Chy

**Figure 1.**
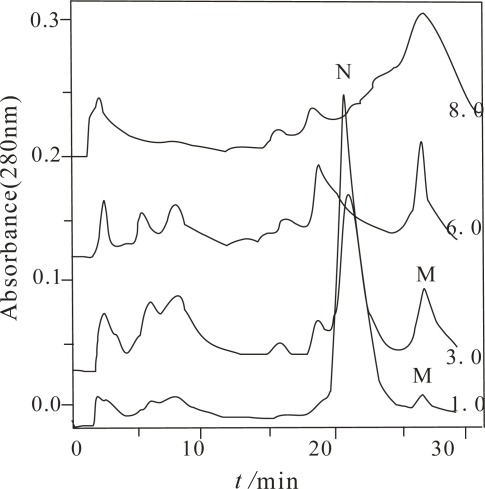
Chromatogram of the denatured α-Chy under different *C*_urea_ by HPHIC. Stationary phase: HIC-silica based PEG-600. Linear gradient elution from 100% solution A, consists of 2.5 mol/L (NH_4_)_2_SO_4_ (pH 7.0) and 0.050 mol/L KH_2_PO_4_ (pH 7.0) to 100% solution B, consists of 0.050 mol/L KH_2_PO_4_ (pH 7.0) for 30 min with a 10 min delay, flow rate: 1.0 mL/min; detection wavelength, 280 nm; N denotes the peak of native α-Chy. M denotes the intermediate state. The number of 1.0, 3.0, 6.0, and 8.0 represents the urea concentration, respectively.

One of advantages of PFLC is that the refolding and separation of proteins can be carried out simultaneously. Based on this advantage, the equilibrium among N, M, U states, even some broken peptide chains, can easily be investigated by measuring the changes in the retention and/or peak height, or by isolating one of them to investigate its character alone, providing a lot of information for understanding the folding mechanism of a target protein. The selected commercial α-Chy is an ideal model protein for accomplishing this purpose. Solid α-Chy in the N state can be bought from commercial sources but it is a kind of self-digesting protein. If one desires to study a feature in a particular state, it has to be prepared from a commercial α-Chy *in situ*. The commercial α-Chy was firstly denatured with various urea concentrations, *C*_urea_ for 24 hours and then was injected into a HIC column for refolding under the same chromatographic conditiond as that for the purification of α-Chy in the N state [16]. Figure 1 only shows the chromatogram of the α-Chy unfolding when *C*_urea_ was 1.0, 3.0, 6.0 and 8.0 mol/L.

From this figure, depending on *C*_urea_, different numbers of peaks were obtained. Compared to other *C*_urea_, the largest number of the components were obtained when *C*_urea_ was 3.0 mol/L. It implies that it is possible for several M states to exist. With the combination of MALDI-TOF MS and bioactivity measurement, only two components have the same molecular mass as the α-Chy, one of them marked with N has high bioactivity and other one, with no bioactivity (actually very low bioactivity) was marked as an M state. When the *C*_urea_ are separately 6.0 and 8.0 mol/L, although the retention times of their last two peaks are the same as the M state at *C*_urea_ 1.0 and 3.0 mol/L, with MALDI-TOF MS identification, they are actually a mixture of many kinds of peptides. This fact indicates a high concentration urea is needed to completely digest the α-Chy. Other components shown in this figure were confirmed to be peptides having various molecular masses. They come either from the commercial product, or from the digestion product during the denaturing and/or renaturating processes. Each of the collected fractions of the α-Chy in the M and N states was lyophilized and stored.

Because the half-life of many intermediates during protein folding is only seconds in duration, sometimes even less, this makes it be very difficult to capture and detect them [17]. The prepared α-Chy in the M state must be stable enough for the subsequent investigations, otherwise, it either is refolded by PFHIC, or self-digested already. The stability of the obtained α-Chy in the M state needs to be initially confirmed by experiment. Re-dissolving each of the lyophilized pellets with either 3.0 mol/L urea (denaturing condition), or water and incubating for 0, 1, 3, 8, 24, and 28 h. The resulting samples were injected into the same HIC column; only one peak having the same retention with the original M state was obtained (Figure not shown here), and also without bioactivity according to the biological assay.

### 4.2. Distinguishing M from U state

This study is required to investigate the character of the α-Chy in the M state, not U state. We firstly need define the protein in M and U states in PFLC. The two states are defined as follows: U state is some species of protein in a non-N state under unfolded conditions, or before PFLC, while the M state is that under refold conditions, or after PFLC. It still needs to be confirmed whether the collection contains to M state or U state. Because the obtained stable α-Chy in the U state exists only under an unfolding condition, i.e., the presence of a suitable urea concentration, otherwise, the U state either refolds to its N state, or converts to a stable M state, or an associated state. In this study, enough urea has to be the present in the sample solution for its identification. In other words, any kind of LC method by which urea from the α-Chy solution is removed, can never be employed to distinguish M from U, or N states.

**Figure 2.**
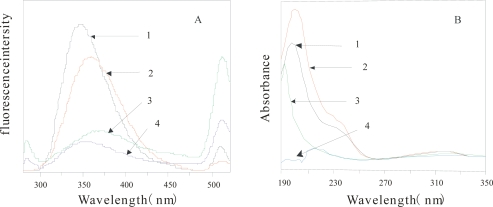
Comparation of FE spectrum and UV absorption spectrometry of the α-Chy in different states. 1. N (C_Urea_=0); 2. U(C_urea_=3.0mol/L); 3. M (C_urea_=0); 4, M (C_urea_=3.0mol/L) (B), UV (195~350nm); (A), FE (250nm). N, native state; M, intermediate state; U, unfold state. C_urea_, urea concentration.

Figure 2 shows the comparison of FE spectrum (2A) and UV absorption spectrometry (2B) of the α-Chy in different environments. From this figure, the two kinds of absorption spectroscopy of U state (2) are quite different from that of M state (3, 4) in either water, or 3.0 mol/L urea solution, also different from N state(1), indicating that the obtained M state(after HIC) shown in Figure 1 is really different from either its U state, or N state.

### 4.3. Z (S) and log I of the α-Chy in N and M states

With isocratic elution, both *Z* and log *I* of proteins in N state can be exactly measured with Equation (1).

**Figure 3.**
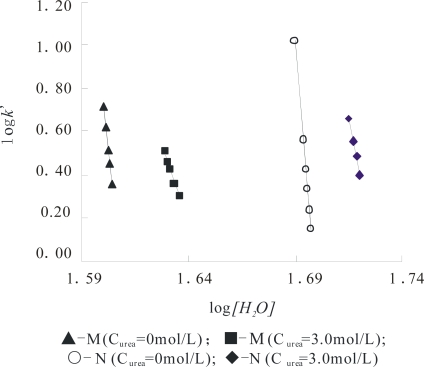
log*k*’ vs. log [*H_2_O*] of the α-Chy in N, M and U states.

For those in an M state, they need testing whether they follow Equation (1), or not. Figure 3 shows this plot of the α-Chy in different states and all parameters for α-Chy in the N and M states at a *C*_urea_ of 0.0 and 3.0 mol/L are separately shown in Table I. It can be seen that although the α-Chy exists in different molecular conformations, the linear correlation coefficient *R* is greater than 0.997, indicating that α-Chy in both of its N and M obeys Equation (1) well. The most relative mean deviation for log *I* and *Z* at two continuously parallel measurements is less than ±3%, providing an experimental basis for characterizing the natures of the N and M states accurately by using both of the log *I* and *Z* values of protein.

### 4.4. Dependence of logI and Z on C_urea_

**Figure 4.**
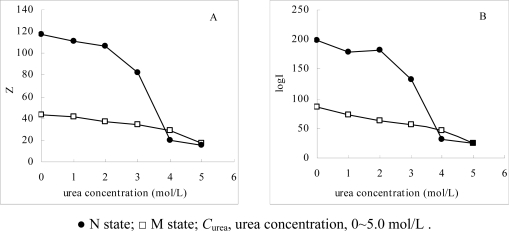
Effect of urea concentration on *Z* (A) and log*I* (B) of α-Chy in N and M states.

Figure 4 shows the effect of *C*_urea_ on log*I* and *Z*, as the *C*_urea_ covers the range from 0 to 5.0 mol/L. It can be seen that the changes in log*I* and *Z* are quite different. For α-Chy as an N state, with the increases in the *C*_urea_, the two values decrease in a tremendous and discontinuous manner, while in that for the M state, they also decrease in the same direction, but only in a small and continuous manner. This phenomenon fits the expectation in the theoretical part that due to the presence of urea the native α-Chy loses significantly its three-, or four-dimensional molecular structure in a discontinuous manner [18]. The part of hydrophobic amino acid residues which are originally buried within the molecules and its surface only has residue with weak hydrophobicity, requiring a large surface area to contact and adsorb by the STHIC. With the increasing the *C*_urea_, more hydrophobic amino residues are exposed to the mobile phase, resulting in contact on the surface of STHIC at a minority of strong hydrophobic amino acid residues. This leads to a decrease in the contact surface area between the α-Chy molecules and the stationary phase in a discontinuous manner. In contrast, because the molecular conformation of α-Chy in the M state had changed a lot already, the changes in log*I* and *Z* are only about a fifth of that in the N state, respectively. This fact indicates that these changes in M state are not a result of molecular conformation changes, but of urea as a secondary displacer (water is the first displacer) to participate in the stoichiometric displacement process [19]. The fact further shows that the α-Chy in the M state is very stable as urea changes, further indicating its M state is very difficult to convert to the N state as urea decreases. It is also seen from Figure 3 that when 3.0 mol/L ≤ *C*_urea_ ≤ 4.0mol/L, the molecular conformation of α-Chy very tremendously changes, and when ≥ 4.0 mol/L, the two values for N state is almost the same as that its M state. Compared to Figure 2 where the optical spectrometry only qualitatively provides the differences between N and M states, the *Z* and log*I* here can provide quantitative information and characterization.

### 4.5. Linear relationship between logI and Z under various C_urea_

**Figure 5.**
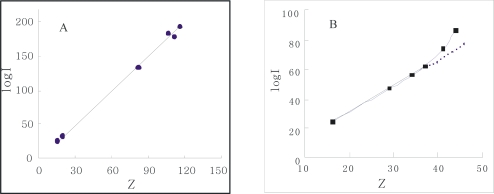
Plot of log*I* vs Z of α-Chy in N (A) and M(B) states.

From Figure 4, the curve profile of the plot of *Z* of α-Chy in N state *vs C*_urea_ has almost the same style as that of of log*I* of α-Chy *vs C*_urea_. The same instance is also seen for α-Chy in M state. It should be tested whether a quantitative relationship really to exist between log *I* and *Z* for a protein in M state as *C*_urea_ changes. It is seen that for its N state shown in Figure 5, a good linear relation with R = 0.9982 exists in Figure 5A and the quantitative expression is indicated by Equation (6). However, for M state, as shown in Figure 5B, there is not this linear relation.

(6) $$\begin{array}{cc} \text{log}I=1.69Z-2.65 & \text{R}=0.9982,\text{N}\;\text{state} \end{array}$$

The obtained *j* value 1.69 is very close to its theoretical value 1.73 (25 °C) [10,12]. This fact indicates that although the changes in molecular conformation of protein in N state are large as *C*_urea_ changes from 0 to 5.0 mol/L, this change is flexible and reversible, resulting in no variation of the non-selective force character between α-Chy molecule and STHIC. In other words, hydrophobic amino acid residues still contact to the STHIC with a moderate hydrophobicity. By contrast, in the same chromatographic condition, the retention of α-Chy in M state is a different situation. Suppose the curve shown in Figure 4B is divided into two parts: (1) the four points continuous on the bottom of this curve which correspond to when *C*_urea_ is 6.0, 5.0, 4.0, 3.0 mol/L; (2) the top four points of this curve when *C*_urea_ is 3.0, 2.0, 1.0, and 0.0 mol/L. It is shown from Figure 4B, the part (1) shows a straight line with R 0.9984 marked with a dash line and it can be expressed by Equation (7):

(7) $$\begin{array}{cc} \text{y}=1.7924\text{x}-4.8479 & \text{R}=0.9984 \end{array}$$

Compared to the *j* value of 1.69 for the N state indicated in Equation (6), the obtained *j* value 1.79 for the M state here is also close to 1.73. The former is 0.04 less than 1.73, indicating the affinity of protein to STHIC (actually the ratio of log*I*/*Z*) becomes weak due to the presence of the second displacer, urea, while the latter is 0.06 greater than 1.73, showing the affinity of the protein to STHIC becomes greater due to the stronger non-selective interaction between them. However, the fact that both close to its theoretical value indicates that the interaction forces between STHIC and α-Chy are totally dominated by the same interaction force. For the non-linear part of the curve shown in Figure 5B, as *C*_urea_ from 0.0~3.0 mol/L (top three points in Figure 5B), α-Chy in the M state interacts with the STHIC to be selective.

This phenomenon suggests that α-Chy in the M state at low urea concentrations tends to refold to its N state and to re-change its molecular structure, making some strong amino acid residues on the surface of α-Chy molecules enter its inside. However, the refolding of α-Chy only proceeds half-way, both hydrophilic and hydrophobic amino acid residues contact to the STHIC and establishes a new equilibrium between non-selective and selective interaction forces at the liquid-solid interface, resulting in not making protein refolding successfully.

## 5. Conclusions

(1) With a hydrophobic interaction chromatography (HIC), a new approach for distinguishing the native (N) state of a protein from its unfold (M) state is presented.

(2) With HIC, a stable M state of the urea-denatured α-chymotrypsin (α-Chy) is prepared and characterized by three parameters of contact surface area (*Z, S*), affinity (log*I*), and the character of interaction force (*j*), of stoichiometric displacement theory for retention (SDT-R).

(3) By comparing the magnitude and type of the three parameters of SDT-R the N and M states some very useful information in proteomics and protein separation in nature may be provided and the existing states of proteins, N, M, U states, may also be distinguished with each other.

## Figures and Tables

**Table 1.** Comparison of *Z* and log *I* of α-Chy between the N and M states.

	*C*_urea_ (mol/L)	*Z*	*S*_1_(%)*	log *I*	*S*_2_(%)*	*R*
**α-Chy (M)**	0 3.0	41.1 34.1	±2.1412 ±0.6891	70.8 56.1	±2.1614 ±0.6687	0.9985 0.9997
Δ*Z* and Δlog*I* (M)	–	–7.0	–	−14.7	–	–
**α-Chy (N)**	0 3.0	117.1 82.4	±0.5952 ±1.5763	198.9 132.5	±0.6093 ±1.5391	0.9987 0.9978
Δ*Z* and Δlog*I* (N)	–	−34.7	–	−66.4	–	–

* , the S_1_ and S_2_ obtained represent the relative mean deviation of values of Z and log I and were measured by two continuous parallel determinations. R is the linear correlation coefficient.
